# Cirrhosis, Age, and Liver Stiffness-Based Models Predict Hepatocellular Carcinoma in Asian Patients with Chronic Hepatitis B

**DOI:** 10.3390/cancers13225609

**Published:** 2021-11-09

**Authors:** Jihye Lim, Young Eun Chon, Mi Na Kim, Joo Ho Lee, Seong Gyu Hwang, Han Chu Lee, Yeonjung Ha

**Affiliations:** 1 Asan Liver Center, Asan Medical Center, University of Ulsan College of Medicine, 88 Olympic-ro 43-gil, Songpa-gu, Seoul 05505, Korea; d140555@amc.seoul.kr; 2 Department of Gastroenterology, CHA Bundang Medical Center, CHA University, 59 Yatap-ro, Bundang-gu, Seongnam-si 13496, Gyeonggi-do, Korea; nachivysoo@chamc.co.kr (Y.E.C.); mina2015@cha.ac.kr (M.N.K.); ljh0505@cha.ac.kr (J.H.L.); sghwang@cha.ac.kr (S.G.H.)

**Keywords:** validation, prediction, CAGE-B, SAGE-B, hepatocellular carcinoma

## Abstract

**Simple Summary:**

Predicting hepatocellular carcinoma in patients with chronic hepatitis B who receive long-term treatment with entecavir or tenofovir is of particular importance in terms of the allocation of medical resources for cancer surveillance. The Cirrhosis and Age (CAGE-B) and Stiffness and Age (SAGE-B) scores were developed to predict hepatocellular carcinoma in Caucasian patients receiving long-term entecavir or tenofovir therapy. In Asian patients who were treated with entecavir or tenofovir, the CAGE-B score predicted the incidence of hepatocellular carcinoma with acceptable accuracy, regardless of the treatment regimen, sex, or hepatic steatosis. Existing prediction models, which showed predictive ability comparable to that of the CAGE-B score, could be used in resource-limited settings where transient elastography is unavailable.

**Abstract:**

Objectives: Predicting hepatocellular carcinoma (HCC) in patients with chronic hepatitis B who received long-term therapy with potent nucleos(t)ide analogs is of utmost importance to refine the strategy for HCC surveillance. Methods: We conducted a multicenter retrospective cohort study to validate the CAGE-B and SAGE-B scores, HCC prediction models developed for Caucasian patients receiving entecavir (ETV) or tenofovir (TFV) for >5 years. Consecutive patients who started ETV or TFV at two hospitals in Korea from January 2009 to December 2015 were identified. The prediction scores were calculated, and model performance was assessed using receiver operating characteristics (ROC) curves. Results: Among 1557 patients included, 57 (3.7%) patients had HCC during a median follow-up of 93 (95% confidence interval, 73–119) months. In the entire cohort, CAGE-B predicted HCC with an area under the ROC curve of 0.78 (95% CI, 0.72–0.84). Models that have “liver cirrhosis” in the calculation, such as AASL (0.79 (0.72–0.85)), CU-HCC (0.77 (0.72–0.82)), and GAG-HCC (0.79 (0.74–0.85)), showed accuracy similar to that of CAGE-B (*p* > 0.05); however, models without “liver cirrhosis”, including SAGE-B (0.71 (0.65–0.78)), showed a lower predictive ability than CAGE-B. CAGE-B performed well in subgroups of patients treated without treatment modification (0.81 (0.73–0.88)) and of male sex (0.79 (0.71–0.86)). Conclusions: This study validated the clinical usefulness of the CAGE-B score in a large number of Asian patients treated with long-term ETV or TFV. The results could provide the basis for the reappraisal of HCC surveillance strategies and encourage future prospective validation studies with liver stiffness measurements.

## 1. Introduction

Hepatitis B virus (HBV) replication results in hepatic inflammation, replacement of normal liver by fibrotic tissue, and progression to cirrhosis, liver failure, and hepatocellular carcinoma (HCC) [1,2,3,4,5]. Therefore, the most fundamental and important strategy for preventing HCC is to suppress the viral replication [6,7].

As a result of treatment with potent nucleos(t)ide analogs (NAs), such as entecavir (ETV) and tenofovir (TFV), the incidence of hepatitis flare and hepatic decompensation has dramatically reduced; however, the risk of HCC cannot be eliminated [1,2,3,4,5]. In particular, predicting HCC in patients whose viral load and hepatic inflammation are well-controlled due to long-term NA therapy is of particular interest [8,9], considering the limited medical resources in many HBV-endemic areas [10,11].

Recently, the Cirrhosis and Age (CAGE-B) and Stiffness and Age (SAGE-B) scores were developed for predicting HCC in Caucasian patients who had been treated with ETV or TFV for at least 5 years due to chronic hepatitis B (CHB) [12]. As the name suggests, the CAGE-B score consists of presence of cirrhosis at baseline and its change during the antiviral therapy, which is assessed using liver stiffness measurements (LSMs) at 5 years, and the patients’ age at 5 years of treatment. The SAGE-B score is a simplified version of CAGE-B, which includes LSM values and age at the 5-year mark of NA therapy.

Little is known about whether these scores can predict the incidence of HCC in Asian patients who are receiving long-term NA therapy with potent antiviral agents. Therefore, we attempted to validate the CAGE-B and SAGE-B scores in patients who had been treated with ETV or TFV for more than 5 years at two university hospitals in South Korea. Moreover, we compared the performance of the scores with those of various HCC prediction models. Finally, subgroup analyses were performed to demonstrate whether the CAGE-B and SAGE-B scores can estimate the risk of HCC in various clinical situations.

## 2. Methods

### 2.1. Study Design

Patients treated with ETV or TFV due to CHB from 1 January 2009 to 31 December 2015 were retrospectively identified from the medical records of two university hospitals in South Korea, namely CHA Bundang Medical Center and Asan Medical Center. Patients treated for less than 5 years or diagnosed with HCC within the first 5 years of treatment were excluded. Additionally, those who had decompensated liver cirrhosis at baseline, who were coinfected with hepatitis C virus, or who had received liver transplantation before or within 5 years after the initiation of NA therapy were excluded.

The diagnoses of liver cirrhosis and HCC were made if one or more of the clinical, imaging, and histological criteria were met. Clinical information, laboratory parameters, and LSM at baseline and at 5 years of treatment were collected.

The Ethical Committees of CHA Bundang Medical Center (approval no. 2021-07-075) and Asan Medical Center (approval no. 2021-1211) approved the study protocol, and written informed consent was waived due to the retrospective nature of the study.

### 2.2. Statistics

The endpoint was the development of HCC beyond 5 years of NA therapy. The Student’s *t*-test or the Mann–Whitney *U*-test was used to compare the characteristics between patients who developed HCC and those who did not, depending on the distribution of continuous variables. The chi-square test was used to compare the categorical variables between the two groups.

The CAGE-B and SAGE-B scores were calculated in each patient using the previously published formula. Additionally, the Age Albumin Sex Liver cirrhosis (AASL), [13] Chinese University (CU)-HCC, [14] Guide with Age, Gender, HBV DNA, Core Promoter Mutations and Cirrhosis (GAG-HCC), [15] Platelet Age Gender (PAGE-B), [16] modified PAGE-B, [17] and Risk Estimation for HCC in CHB (REACH-B) [18] scores were also calculated for comparisons.

The performance of various HCC prediction models was assessed using receiver operating characteristics (ROC) curves. The area under the ROC curve (AUC) and corresponding 95% confidence intervals (CIs) were calculated and compared using the DeLong test. Additionally, standard measures of predictive accuracy, including sensitivity, specificity, positive predictive value, and negative predictive value, were used to evaluate the predictive performance of each model.

Subgroup analyses involving patients whose NA regimen had not been changed during the study period, those who were treated with ETV or TFV, those of the male sex, and those with hepatic steatosis at baseline and at 5 years of treatment were subsequently performed. Hepatic steatosis was defined based on the controlled attenuation parameter value of ≥238 dB/m.

The SPSS (version 26.0), R (version 4.0.5), and R Studio (version 4.1106), including the pROC package, were used for data analyses. In accordance with ref. [19], *p*-values of less than 0.05 were used to denote statistical significance.

## 3. Results

### 3.1. Study Cohort and Patient Characteristics

The study cohort consisted of 1557 patients who received ETV (*n* = 868 (55.7%)) or TFV (*n* = 689 (44.3%)) for treatment of CHB between 1 January 2009 and 31 December 2015. The clinical characteristics of all included patients are shown in Table 1.

The median duration of follow-up was 93 months (interquartile range (IQR), 73–119 months) and 57 patients (3.7%) were diagnosed with HCC during the study period. Patients who were diagnosed with HCC (HCC group) were significantly older (51 vs. 46 years, *p* = 0.002), had more cirrhosis (75.4% vs. 25.9%; *p* < 0.001), and had higher LSM values (17.9 kPa vs. 7.3 kPa; *p* < 0.001) at baseline than those without HCC (no-HCC group). The proportion of patients treated with TFV was significantly higher in the no-HCC group (45.1%) than that in the HCC group (22.8%; *p* = 0.001). In patients whose aspartate aminotransferase (AST) and alanine aminotransferase (ALT) levels were higher than 40 IU/L at treatment initiation, the median AST and ALT levels were significantly higher in the no-HCC group (*P*s < 0.05). In contrast, the median platelet count was significantly lower in the HCC group than that in the no-HCC group (127 × 1000 vs. 168 × 1000/mm^3^; *p* < 0.001). Albumin, total bilirubin, and prothrombin time levels were also significantly different between the two groups, although the median values were within normal limits. The hepatitis B e antigen (HBeAg) positivity and HBV DNA titers did not differ between the two groups.

At 5 years of treatment, the LSM values and AST levels were significantly higher in the HCC groups than in the no-HCC group (*P*s < 0.05), whereas the median platelet counts were lower (144 × 1000 vs. 190 × 1000/mm^3^; *p* < 0.001). The proportion of patients with HBeAg seroconversion or undetectable HBV DNA at 5 years was not significantly different between the two groups.

### 3.2. Performance of CAGE-B, SAGE-B, and Other Prediction Models

**Figure 1:**
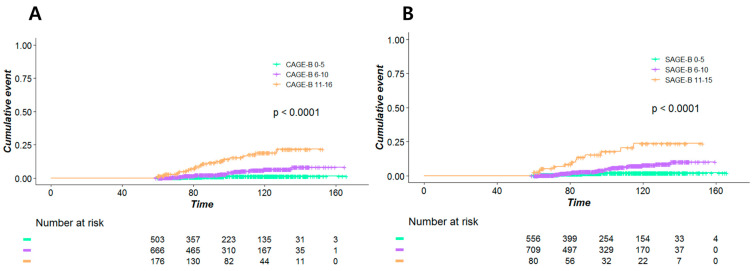
Kaplan–Meier estimates of the incidence of hepatocellular carcinoma according to the (**A**) CAGE-B and (**B**) SAGE-B risk groups.

Our patients were classified into three groups according to the calculated CAGE-B and SAGE-B scores. Figure 1A reveals that the high-CAGE-B-score group had a significantly higher incidence of HCC than the intermediate- or low-CAGE-B-score group (*p* < 0.001). Similarly, the incidence of HCC showed significant trichotomization according to the SAGE-B scores (Figure 1B).

**Figure 2:**
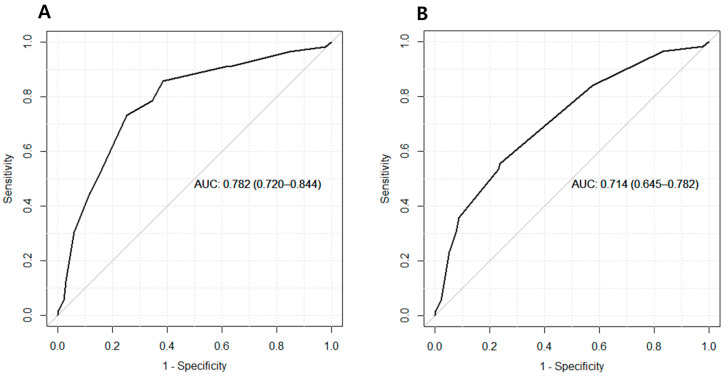
Area under the receiver operating characteristics curve of (**A**) the CAGE-B and (**B**) SAGE-B scores for predicting hepatocellular carcinoma.

Subsequently, the performance of HCC prediction scores, including CAGE-B and SAGE-B, was assessed. The CAGE-B score detected HCC with an AUC of 0.78 (95% CI, 0.72–0.84; Table 2). This corresponded to a sensitivity of 0.73 and a specificity of 0.75. Meanwhile, the AUC, sensitivity, and specificity were 0.71 (95% CI, 0.65–0.78), 0.55 and 0.76, respectively, for the SAGE-B score. The AUC of the CAGE-B score was significantly higher than that of the SAGE-B score (DeLong *p* < 0.001). The ROC curves are shown in Figure 2.

**Figure 3:**
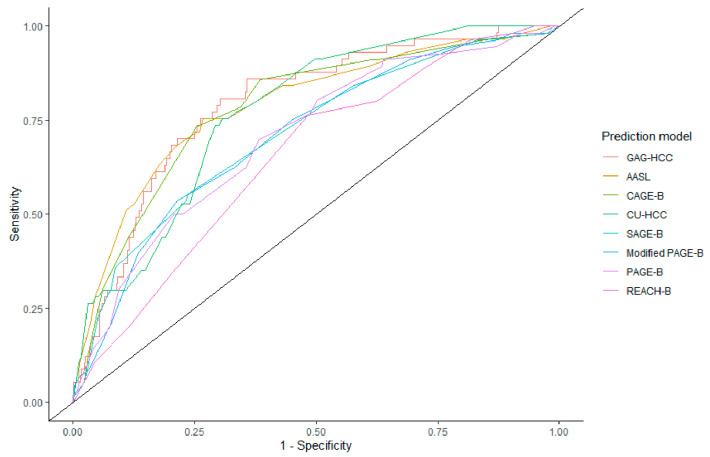
Area under the receiver operating characteristics curve of hepatocellular carcinoma prediction models.

Prediction models that were developed for the Asian cohort also showed high performance, with an AUC of 0.79 (0.72–0.85) for AASL, 0.77 (0.72–0.82) for CU-HCC, and 0.79 (0.74–0.85) for GAG-HCC. The AUCs of these prediction models did not show significant differences to that of CAGE-B (DeLong *p* > 0.05). In contrast, the AUCs of PAGE-B, modified PAGE-B, and REACH-B were lower than that of the CAGE-B score. The ROC curves of each prediction model are shown in Figure 3. The sensitivity and specificity of AASL, CU-HCC, and GAG-HCC were comparable to those of CAGE-B. The positive and negative predictive values were comparable across the prediction models (Table 2).

### 3.3. Subgroup Analysis

We tested whether CAGE-B, SAGE-B, and other prediction models were valid in different clinical contexts by performing subgroup analyses.

#### 3.3.1. Patients without Treatment Modification

**Figure 4:**
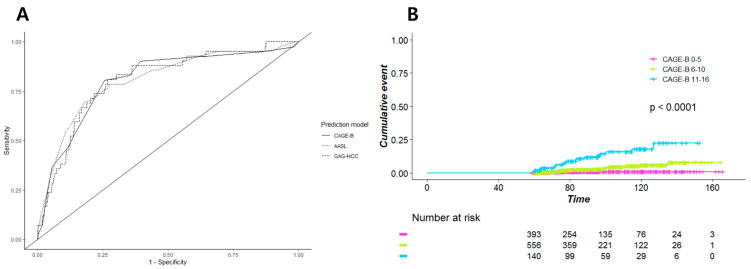
(**A**) Area under the receiver operating characteristics curve of the CAGE-B, AASL, and GAG-HCC scores and (**B**) Kaplan–Meier estimates of the incidence of hepatocellular carcinoma according to the CAGE-B risk group in the subgroup of patients who received entecavir or tenofovir without treatment modification.

Of the 1557 patients, 1295 (83.2%) received ETV or TFV throughout the study period without changes in the NA regimen. In these patients, the AUC for HCC vs. no HCC was 0.81 (95% CI, 0.73–0.88) for the CAGE-B score, which was significantly higher than that of SAGE-B, PAGE-B, modified PAGE-B, and REACH-B (Table 3). The AUCs of AASL, CU-HCC, and GAG-HCC were not significantly different from that of CAGE-B (Figure 4A). However, when we compared the two models with the highest AUCs, CAGE-B and AASL, using Kaplan–Meier estimates, the CAGE-B score showed better differentiation between the three risk groups (Figure 4B). Moreover, the CAGE-B score performed well in predicting HCC in both the ETV- and TFV-treated groups (Table 3).

#### 3.3.2. Male Patients

The CAGE-B score showed the highest AUC value in male patients (*n* = 993) with GAG-HCC (Supplementary Figure S1). However, similar to the results in the entire cohort, no statistically significant difference among CAGE-B, GAG-HCC, AASL, and CU-HCC was observed. The predictive ability of the CAGE-B score was higher than that of PAGE-B, modified PAGE-B, and REACH-B in male patients (Table 3).

#### 3.3.3. Patients with Hepatic Steatosis

Hepatic steatosis was defined using the controlled attenuation parameter (CAP) value. If the CAP value was higher than 238 dB/m, patients were considered to have hepatic steatosis. It was found that 155 and 567 patients had hepatic steatosis at baseline and 5 years of treatment, respectively. In patients with hepatic steatosis at baseline, a statistical significance was not identified among the eight prediction models, despite the difference in AUC values (Table 3). In patients with hepatic steatosis at the 5-year mark, the AUC of the CAGE-B score was 0.78 (95% CI, 0.67–0.90); however, it was significantly lower than that of Asian prediction models, that is, AASL, CU-HCC, and GAG-HCC. The AUCs of AASL, CU-HCC, and GAG-HCC were statistically comparable in this subgroup. In particular, the CU-HCC score showed a better discriminative ability (Supplementary Figure S2A) than the AASL score (Supplementary Figure S2B).

## 4. Discussion

In this multicenter retrospective study involving patients treated with ETV or TFV due to CHB, we showed for the first time that the CAGE-B score, composed of cirrhosis status at baseline, LSM value at 5 years of treatment, and age at 5 years, can successfully predict HCC with acceptable accuracy (AUC of higher than 0.75) in patients receiving long-term NA therapy. Furthermore, the CAGE-B score performed well in subgroups of patients who received ETV or TFV throughout the study period without treatment modification, in male patients, and in those with hepatic steatosis at the 5-year mark of NA therapy.

As a result of the NA therapy, HBV replication, hepatic inflammation, progression to fibrosis and cirrhosis, and the development of decompensation and HCC have been dramatically reduced [1,2,3,4,5]. However, even a durable viral suppression by long-term therapy with potent NAs, such as ETV and TFV, cannot eliminate the risk of HCC [1,2,3,4,5]. The CAGE-B and SAGE-B scores were developed to predict HCC in this special population with well-controlled viremia, using the data of Caucasian patients with CHB who had been receiving ETV or TFV for more than 5 years [12]. Although both scores performed well in Caucasian patients, the CAGE-B score performed better than the SAGE-B score in this Asian validation study. The only difference between the two scores is that SAGE-B does not have “liver cirrhosis at baseline” in the calculation of the score.

Traditionally, liver cirrhosis is considered an irreversible condition and, therefore, has been regarded as a well-known risk factor for HCC, irrespective of the etiologies of underlying liver disease [1,2]. Therefore, evaluating whether patients have liver cirrhosis or not is essential. However, due to the limitations inherent to noninvasive tests, accurately defining the presence of cirrhosis is often difficult. Particularly in patients with macronodular and/or inactive cirrhosis, such as those with CHB and well-controlled viremia, LSM alone can underestimate the actual cirrhosis status [20,21,22,23]. Additionally, studies have identified that genotype C, which is the most prevalent HBV genotype in Korea, was associated with more active hepatitis, advanced liver disease, and HCC [24,25]. Based on these studies and our results, we think that using the SAGE-B score over other prediction models including CAGE-B is rather premature because SAGE-B can miss patients who are at risk of HCC due to macronodular/inactive cirrhosis and aggressive HBV genotype. Actually, the median LSM value of the 39 patients who developed HCC despite low LSM values at 5 years (<12 kPa) was 7.1 kPa (IQR, 5.4–8.9 kPa) in this study. The value is still high compared with the measurements from patients without HCC (Table 1, median 4.8 kPa, IQR 3.9–6.4). Therefore, although LSM correlates well with the fibrosis stage [22,23], baseline cirrhotic status should also be considered for risk stratification in Asian patients.

The CAGE-B score performed well in subgroup analyses. In patients who were continuously treated with ETV or TFV without treatment modifications, the CAGE-B score showed excellent discrimination with an AUC of 0.81 and a clear split of the incidence curves according to the risk group. This result was reproduced in ETV- or TFV-treated subsets, and in the male subgroup. However, we found the CAGE-B score to be less discriminative when applied to patients with CAP value-based fatty liver. In patients who had CAP values of higher than 238 dB/m at baseline, all prediction models showed similar predictive performance. This could be, at least partly, attributed to the small number of patients in this subgroup (*n* = 155). In contrast, in patients who had fatty liver at the 5-year mark, the CAGE-B score differentiated those who developed HCC from those who did not with acceptable accuracy. However, the predictive performance of the AASL, CU-HCC, and GAG-HCC scores was significantly better than that of CAGE-B. Asians are less obese than Caucasians, and additionally, the AASL, CU-HCC, and GAG-HCC scores have “albumin” in common as a component of the scoring system. Therefore, it is plausible that obesity and nutritional status are factors that are attributable to the lower predictability of the CAGE-B score in patients with high CAP values. Further studies analyzing the impact of fatty liver disease, obesity, or nutritional status on the accuracy of the CAGE-B score would be needed.

Of note, the AASL, CU-HCC, and GAG-HCC scores showed a performance comparable to that of CAGE-B in the entire cohort, and in subgroups of patients without treatment modifications or in those with male sex. The AASL score consisted of age, albumin, sex, and liver cirrhosis [13]. The components of CU-HCC are age, albumin, liver cirrhosis, bilirubin, and HBV DNA [14]. GAG-HCC also had age, liver cirrhosis, and HBV DNA, along with sex [15]. These three models have age and liver cirrhosis in common and other components including albumin, bilirubin, and HBV DNA levels can be easily obtained during standard patient care [1,2]. Therefore, the aforementioned prediction models could also be used for predicting HCC beyond 5 years of potent NA therapy, particularly in HBV-endemic regions with limited medical resources, although these models were not developed for that aim.

The strength of this study is that we analyzed a large number of homogeneous patients who were treated uniformly with ETV or TFV for at least for 5 years at two university hospitals. A recent study conducted in South Korea attempted to validate the CAGE-B and SAGE-B scores in Asian patients; however, those treated with other NAs, besides ETV or TFV, or those treated for less than 5 years were included in that study [26]. In our validation study, which included patients who were essentially the same as the original CAGE-B/SAGE-B cohort, the CAGE-B score yielded an acceptable predictive accuracy with AUCs varying between 0.75 and 0.85 in the entire and subgroup analyses. Of note, we reported that the SAGE-B score and other prediction models that do not have “liver cirrhosis at baseline” as a component of scoring system had lower predictive ability. This finding suggests that baseline cirrhosis status should be considered an important predictor of future HCC development in patients who have macronodular/inactive cirrhosis despite long-term NA therapy, particularly if they have a genotype C HBV infection. Additionally, the AASL, CU-HCC, and GAG-HCC scores constitute potential alternatives to the CAGE-B score in HBV-endemic areas where advanced medical resources such as transient elastography are limited [10,11].

A potential weakness of this study lies in its retrospective nature. First, LSM was performed at the discretion of the managing physicians. Therefore, we could not calculate the CAGE-B or SAGE-B scores in patients without the data, particularly those who started NA therapy before 2012 when transient elastography was first introduced at the institution(s) that participated in this study. Nevertheless, 85.9% of the patients had LSM data at 5 years of NA therapy, and we attempted to enhance the robustness of our results by performing subgroup analyses. Future prospective longitudinal studies that obtain LSM data on a regular basis, particularly at 5 years of NA therapy, could provide more comprehensive evidence for utilizing CAGE-B scores in clinical practice.

## 5. Conclusions

We validated the CAGE-B and SAGE-B scores in Asian patients who were treated with ETV or TFV for more than 5 years. The CAGE-B score, which consists of baseline cirrhosis status, age, and LSM at 5 years, successfully discriminated patients with HCC from those without HCC beyond 5 years of treatment. Our validation study could be a foundation for the reappraisal of future HCC surveillance strategies in Asian patients.

## Figures and Tables

**Table 1:** Cohort characteristics at baseline and 5 years of treatment *.

Variable	All (*n* = 1557)	No HCC (*n* = 1500)	HCC (*n* = 57)	*p*-Value
Baseline				
Age, years	46.6 ± 10.6	46.4 ± 10.6	50.9 ± 9.6	0.002
Sex				0.21
Male	993 (63.8)	952 (63.5)	41 (71.9)	
Female	564 (36.2)	548 (36.5)	16 (28.1)	
Cirrhosis	431 (27.7)	388 (25.9)	43 (75.4)	<0.001
LSM, kPa	7.4 (4.8, 12.3)	7.3 (4.8, 12.0)	17.9 (10.9, 26.3)	<0.001
Initial nucleos(t)ide analog				0.001
Entecavir	868 (55.7)	824 (54.9)	44 (77.2)	
Tenofovir	689 (44.3)	676 (45.1)	13 (22.8)	
Albumin, g/dL	4.2 (3.8, 4.4)	4.2 (3.8, 4.4)	4.0 (3.3, 4.3)	0.004
Fasting glucose, mg/dL	98.0 (91.0, 108.0)	98.0 (91.0, 108.0)	105.0 (90.8, 120.5)	0.09
Creatinine, mg/dL	0.9 (0.7, 1.0)	0.9 (0.7, 1.0)	0.9 (0.7, 1.0)	0.78
Total bilirubin, mg/dL	0.9 (0.6, 1.2)	0.8 (0.6, 1.2)	1.1 (0.7, 1.7)	0.001
AST, IU/L	53.0 (30.0, 106.8)	53.0 (30.0, 107.0)	53.0 (43.0, 107.5)	0.15
AST in patients with AST > 40 IU/L	86.0 (56.0, 157.5)	86.5 (57.0, 159.0)	57.0 (47.0, 133.0)	0.016
ALT, IU/L	57.0 (28.0, 130.0)	58.0 (27.0, 130.0)	51.0 (37.5, 112.5)	0.69
ALT in patients with AST > 40 IU/L	105.0 (62.0, 202.0)	106.5 (63.0, 202.8)	76.0 (47.8, 146.0)	0.017
ALP, IU/L	93.0 (67.0, 155.8)	92.5 (67.0, 153.8)	116.0 (64.0, 184.0)	0.26
GGT, U/L	46.0 (23.0, 109.0)	44.5 (22.0, 108.0)	67.0 (36.5, 128.0)	0.011
Platelet, ×1000/mm^3^	166 (126, 204)	168 (128, 206)	127 (80, 160)	<0.001
Prothrombin time, INR	1.1 (1.0, 1.1)	1.1 (1.0, 1.1)	1.1 (1.0, 1.4)	0.001
HBeAg positivity	830 (60.5)	802 (60.8)	28 (50.9)	0.16
HBV DNA, log IU/mL	5.8 (3.5, 7.4)	5.8 (3.4, 7.4)	6.1 (4.7, 7.2)	0.23
HBV DNA in patients with detectable HBV DNA, log IU/mL	6.1 (4.4, 7.6)	6.2 (4.4, 7.6)	6.4 (4.8, 7.2)	0.79
At 5 years of treatment				
LSM, kPa	4.9 (4.0, 6.7)	4.8 (3.9, 6.4)	8.8 (6.4, 13.0)	<0.001
AST, IU/L	24.0 (20.0, 29.0)	24.0 (20.0, 29.0)	28.0 (22.0, 37.5)	0.001
ALT, IU/L	20.0 (14.0, 28.0)	20.0 (14.0, 28.0)	22.0 (16.5, 32.5)	0.12
Platelet, ×1000/mm^3^	189 (151, 228)	190 (154, 230)	144 (94, 174)	<0.001
HBeAg seroconversion	319 (41.3)	304 (40.8)	15 (55.6)	0.16
Undetectable HBV DNA	1393 (91.0)	1345 (91.3)	48 (84.2)	0.09
Follow-up duration, months	92.8 (72.7, 119.3)	92.0 (72.7, 118.7)	113.6 (85.7, 129.6)	<0.001

* Data are mean ± standard deviation, median (interquartile ranges), or *n* (%). HCC, hepatocellular carcinoma; LSM, liver stiffness measurement; AST, aspartate aminotransferase; ALT, alanine aminotransferase; ALP, alkaline phosphatase; INR, international normalized ratio; HBeAg, hepatitis B e antigen; HBV, hepatitis B virus.

**Table 2:** Performance characteristics of hepatocellular carcinoma prediction models.

	CAGE-B	SAGE-B	AASL	CU-HCC	GAG-HCC	PAGE-B	Modified PAGE-B	REACH-B
AUC (95% CI)	0.78 (0.72–0.84)	0.71 (0.65–0.78)	0.79 (0.72–0.85)	0.77 (0.72–0.82)	0.79 (0.74–0.85)	0.71 (0.64–0.77)	0.71 (0.64–0.78)	0.65 (0.59–0.72)
Sensitivity (LL–UL)	0.73 (0.60–0.83)	0.55 (0.42–0.68)	0.75 (0.63–0.86)	0.75 (0.63–0.86)	0.81 (0.67–0.89)	0.70 (0.55–0.81)	0.54 (0.39–0.52)	0.76 (0.64–0.86)
Specificity (LL–UL)	0.75 (0.63–0.80)	0.76 (0.64–0.85)	0.73 (0.54–0.82)	0.69 (0.57–0.74)	0.70 (0.46–0.79)	0.62 (0.48–0.70)	0.79 (0.63–0.85)	0.52 (0.32–0.60)
PPV	0.11	0.09	0.10	0.09	0.09	0.07	0.09	0.06
NPV	0.98	0.98	0.99	0.99	0.99	0.98	0.98	0.98
Cutoff	5, 10	5, 10	5, 19	5, 20	100	9, 17	8, 12	7

AUC, area under the curve; CI, confidence interval; LL, lower limit; UL, upper limit; PPV, positive predictive value; NPV, negative predictive value.

**Table 3:** Performance characteristics of hepatocellular carcinoma prediction models in subgroups of patients.

		AUC (95% CI)							
	Prediction Model	CAGE-B	SAGE-B	AASL	CU-HCC	GAG-HCC	PAGE-B	Modified PAGE-B	REACH-B
Subgroup									
No change in treatment regimen (*n* = 1295)		0.81 (0.73–0.88)	0.73 (0.64–0.81)	0.81 (0.73–0.88)	0.78 (0.72–0.84)	0.81 (0.74–0.87)	0.72 (0.64–0.80)	0.71 (0.64–0.78)	0.66 (0.58–0.74)
TFV (*n* = 680)		0.72 (0.52–0.93)	0.65 (0.44–0.85)	0.67 (0.48–0.86)	0.71 (0.59–0.82)	0.70 (0.52–0.88)	0.71 (0.55–0.87)	0.66 (0.47–0.84)	0.73 (0.60–0.87)
ETV (*n* = 615)		0.82 (0.76–0.89)	0.75 (0.66–0.84)	0.85 (0.79–0.91)	0.79 (0.72–0.87)	0.83 (0.78–0.89)	0.74 (0.65–0.82)	0.74 (0.66–0.83)	0.63 (0.52–0.73)
Male (*n* = 993)		0.79 (0.71–0.86)	0.72 (0.64–0.80)	0.77 (0.69–0.85)	0.78 (0.72–0.84)	0.79 (0.73–0.85)	0.73 (0.66–0.80)	0.71 (0.63–0.78)	0.64 (0.55–0.72)
CAP at baseline ≥ 238 dB/m (*n* = 155)		0.71 (0.47–0.95)	0.62 (0.37–0.87)	0.74 (0.47–1.00)	0.77 (0.63–0.91)	0.74 (0.48–0.99)	0.71 (0.49–0.93)	0.63 (0.38–0.88)	0.66 (0.49–0.84)
CAP at 5-year ≥ 238 dB/m (*n* = 567)		0.78 (0.67–0.90)	0.68 (0.55–0.80)	0.83 (0.72–0.94)	0.83 (0.77–0.89)	0.85 (0.75–0.94)	0.74 (0.63–0.84)	0.71 (0.59–0.83)	0.65 (0.54–0.75)

AUC, area under the curve; CI, confidence interval; TDF, tenofovir disoproxil fumarate; CAP, controlled attenuation parameter.

## Data Availability

The datasets used and/or analyzed during this study are available from the corresponding author upon reasonable request.

## Supplementary Materials

The following are available online at https://www.mdpi.com/article/10.3390/cancers13225609/s1, Figure S1: Area under the receiver operating characteristics curve of CAGE-B and GAG-HCC in male patients, Figure S2: Kaplan–Meier estimates of the incidence of hepatocellular carcinoma according to the (**A**) CU-HCC and (**B**) AASL risk groups in the subgroup of patients who had fatty liver at 5 years of nucleos(t)ide analog therapy.

## Abbreviations

HBV, hepatitis B virus; HCC, hepatocellular carcinoma; NA, nucleos(t)ide analog; ETV, entecavir; TFV, tenofovir; CAGE-B, Cirrhosis and Age; SAGE-B, Stiffness and Age; CHB, chronic hepatitis B; LSM, liver stiffness measurement; AASL, Age Albumin Sex Liver cirrhosis; CU, Chinese University; GAG-HCC, Guide with Age, Gender, HBV DNA, Core Promoter Mutations and Cirrhosis; PAGE-B, Platelet Age Gender; REACH-B, Risk Estimation for HCC in CHB; ROC, receiver operating characteristics; AUC, area under the ROC curve; CI, confidence interval; IQR, interquartile ranges; AST, aspartate aminotransferase; ALT, alanine aminotransferase; HBeAg, hepatitis B e antigen.
