# Supplementary material for: Cirrhosis, Age, and Liver Stiffness-Based Models Predict Hepatocellular Carcinoma in Asian Patients with Chronic Hepatitis B
Source: Cancers (Basel). 2021 Nov 9;13(22):5609. doi: 10.3390/cancers13225609 (PMC8615754; doi:10.3390/cancers13225609)
Supplement: Supplementary file 1 [file cancers-13-05609-s001.zip › cancers-1435760-supplementary.pdf]

Supplementary Material

Cirrhosis, Age, and Liver Stiffness-Based Models Predict Hepatocellular Carcinoma in Asian Patients with Chronic Hepatitis B

Jihye Lim, Young Eun Chon, Mi Na Kim, Joo Ho Lee, Seong Gyu Hwang, Han Chu Lee and Yeonjung Ha

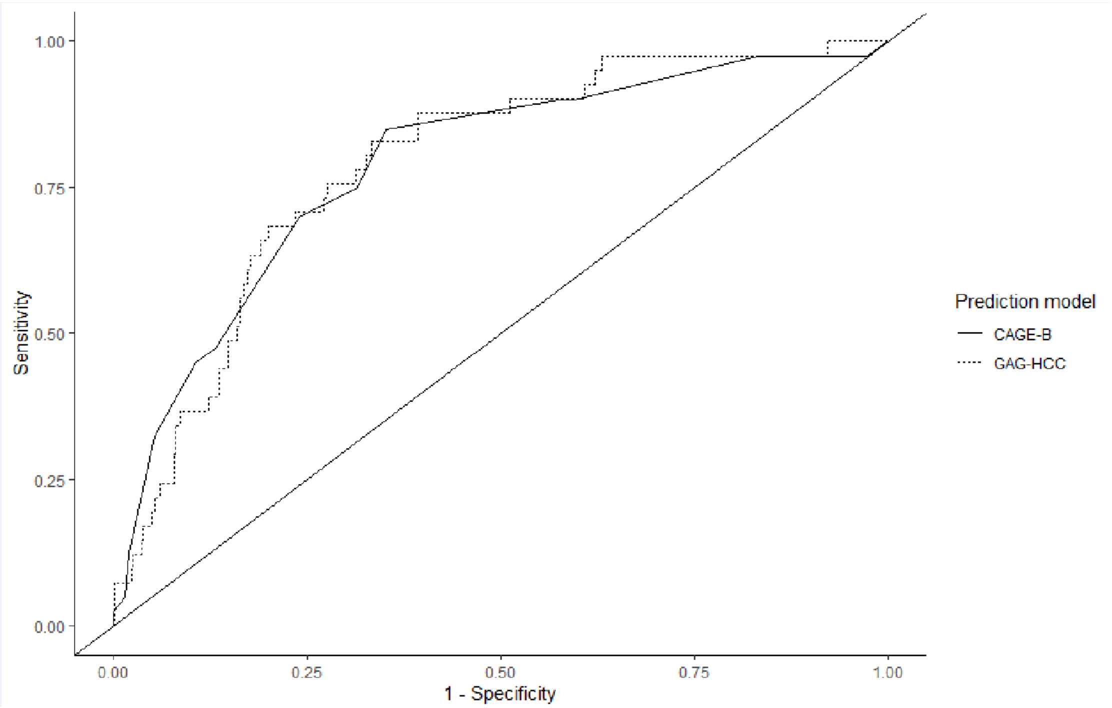

Figure S1. Area under the receiver operating characteristics curve of CAGE-B and GAG-HCC in male patients.

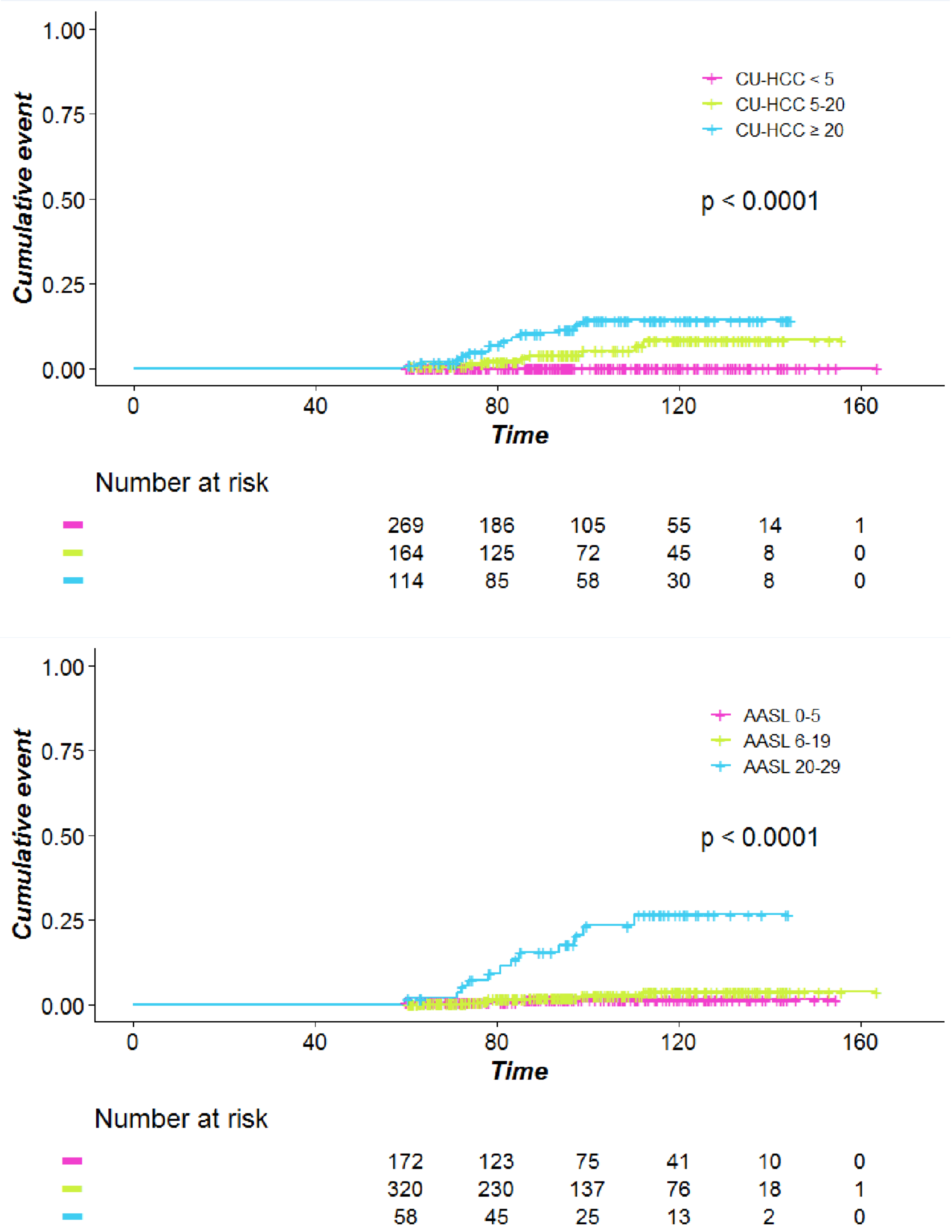

Figure S2. Kaplan-Meier estimates of the incidence of hepatocellular carcinoma according to the (**A**) CU-HCC and (**B**) AASL risk groups in the subgroup of patients who had fatty liver at 5 years of nucleos(t)ide analog therapy.
